# Exploring the prevalence of undetected bradyarrhythmia in dementia with Lewy bodies

**DOI:** 10.1007/s10286-023-00962-w

**Published:** 2023-07-05

**Authors:** Isak Heyman, Torbjörn Persson, Mattias Haglund, Elisabet Londos

**Affiliations:** 1 Cognitive Disorder Research Unit, Department of Clinical Sciences Malmö, Lund University, Malmö, Sweden; 2 Department of Cardiology, Skane University Hospital, Malmö, Sweden; 3 Division of Pathology, Department of Clinical Sciences Lund, Lund University, Lund, Sweden; 4 Division of Clinical Geriatrics, Department of Neurobiology, Care Sciences and Society, Karolinska Institute, Stockholm, Sweden

**Keywords:** Dementia with Lewy bodies, Ambulatory electrocardiographic monitoring, Bradyarrhythmia, Sick sinus syndrome

## Abstract

**Purpose:**

To explore the prevalence of undetected bradyarrhythmia in a cohort of people with dementia with Lewy bodies.

**Methods:**

Thirty participants diagnosed with dementia with Lewy bodies were enrolled from three memory clinics in southern Sweden between May 2021 and November 2022. None had a history of high-grade atrioventricular block or sick sinus syndrome. Each participant underwent orthostatic testing, cardiac [^123^I]metaiodobenzylguanidine scintigraphy and 24-h ambulatory electrocardiographic monitoring. Concluding bradyarrhythmia diagnosis was obtained until the end of December 2022.

**Results:**

Thirteen participants (46.4%) had bradycardia at rest during orthostatic testing and four had an average heart rate < 60 beats per minute during ambulatory electrocardiographic monitoring. Three participants (10.7%) received a diagnosis of sick sinus syndrome, of whom two received pacemaker implants to manage associated symptoms. None received a diagnosis of second- or third-degree atrioventricular block.

**Conclusion:**

This report showed a high prevalence of sick sinus syndrome in a clinical cohort of people with dementia with Lewy bodies. Further research on the causes and consequences of sick sinus syndrome in dementia with Lewy bodies is thus warranted.

## Introduction

Dementia with Lewy bodies (DLB) and Parkinson’s disease (with and without dementia) are incurable clinical entities caused by neurotoxic deposits of the intracellular protein, alpha-synuclein (α-syn) [1]. DLB and Parkinson’s disease are often referred to as Lewy body disease (LBD), with DLB reported as the second most common subtype of neurodegenerative dementia worldwide [2].

The spreading of α-syn is thought to propagate centripetally, starting in autonomic nerves before reaching the central nervous system [3]. Furthermore, deposits of α-syn are commonly found in cardiac nervous tissue [4–6], as well as in the sinus node [7]. Cardiac Lewy body pathology can be present even when involvement of α-syn in the central nervous system is limited [4]. Additionally, an association between cardiac Lewy body pathology and cardiac sympathetic denervation has been suggested [8, 9], with cardiac sympathetic denervation being common among people with LBD [10].

Authors of previous research have proposed that cardiac Lewy body pathology might manifest as arrhythmia [7, 11]. Impaired function of the sinus node and/or cardiac conduction system may cause symptomatic bradycardia, such as high-grade atrioventricular (AV) block or sick sinus syndrome (SSS) [12, 13]. A recent clinicopathological study found that 3.9% and 5.2% of patients with confirmed LBD had third-degree AV block and SSS, respectively [14]. For comparison, in the general population, third-degree AV block and SSS have a prevalence of 0.04% and 0.17%, respectively [15, 16]. To our knowledge, no other studies have investigated the prevalence of bradyarrhythmia among people with DLB.

In clinical practice, symptoms of bradycardia are non-specific and may include light-headedness, fatigue and presyncope/syncope, and may also cause dementia-like symptoms due to reduced cerebral blood flow [17]. Thus, SSS has been described as mimicking the features of both DLB and Parkinson’s disease [18–20] and might therefore be an underdiagnosed concurrent syndrome.

One of the salient features of DLB is an increased risk of falls, which is greater in DLB than with Alzheimer’s disease (AD) [21]. Patients with DLB are at high risk of hospitalization [22] compared with patients with AD, and falls have been reported to be the second most common reason for hospitalization in patients with DLB [23]. A significant contributor to falls in the elderly population is orthostatic hypotension (OH) [24], and there are conflicting data as to whether OH is more common in DLB than in AD [25–27]. While several factors likely contribute to the increased risk of falls in DLB, preliminary data suggesting a high prevalence of arrhythmia and a recent case report [20] raise the possibility that arrhythmias, including SSS, could be a potentially modifiable risk factor to reduce autonomic symptoms and falls in patients with DLB.

The present study was undertaken to explore the prevalence of undetected bradyarrhythmia in people with DLB.

## Methods

### Participants

Thirty participants were enrolled from three memory clinics in southern Sweden from May 2021 to November 2022. For inclusion, each participant had to be diagnosed with possible or probable DLB [28]. The presence of cardiac implantable electronic devices constituted an exclusion criterion. Each enrolled participant underwent orthostatic testing, cardiac [^123^I]metaiodobenzylguanidine (MIBG) scintigraphy, and ambulatory electrocardiographic (AECG) monitoring.

Demographics such as cardiovascular diseases, arrhythmia, cardiovascular risk factors, and prescribed medications were obtained from medical records. Cerebrospinal fluid markers, clinical core features of DLB, and indicative biomarkers of DLB were thoroughly assessed as part of routine clinical workup. Mini-Mental State Examination (MMSE), Montreal Cognitive Assessment (MoCa), and Rowland Universal Dementia Assessment Scale (RUDAS) scores at the time of study inclusion were collected.

All participants gave their informed consent prior to their inclusion in the study. The study was approved by the National Ethics Committee of Sweden (No. 2021-01765), and the Regional Ethics Review Board of Scania, Sweden (No. 195-21). The study was conducted in accordance with the Declaration of Helsinki and its later amendments.

### Orthostatic testing

A 3-min orthostatic test was performed by a nurse at the affiliated memory clinic. Participants had to rest for 10 min in a supine position with blood pressure and corresponding palpatory heart rate (HR) measured after resting, directly at active standing, and following 1 and 3 min of active standing. Bradycardia was defined as a resting HR < 60 beats per minute (bpm) [29].

OH was considered if systolic blood pressure (SBP) decreased ≥ 20 mmHg or diastolic BP (DBP) decreased ≥ 10 mmHg within 3 min of active standing [30]. In the presence of supine hypertension (≥ 160 mmHg at rest), a decrease in SBP of ≥ 30 mmHg was considered to be OH [30]. To distinguish the presence of non-neurogenic OH from neurogenic OH (nOH), a ∆HR/∆SBP ratio of < 0.49 at 3 min of active standing was used [31].

### Cardiac MIBG scintigraphy

Cardiac MIBG scintigraphy was performed to assess the presence of cardiac sympathetic denervation. A computer tomography camera was used to obtain images 4 h after intravenous administration of MIBG. Delayed heart/mediastinum ratio (HMR) was calculated as a fraction of the mean count per pixel in the heart region of interest divided by that of the upper mediastinum region of interest. Cardiac sympathetic denervation was defined as a delayed HMR < 1.6 [32]. Neither homogeneity of regions of interest nor MIBG washout rate was addressed in the nuclear medicine reports.

### Electrocardiogram recording

A 10-s resting electrocardiogram (ECG) was conducted for each participant before AECG monitoring started. Ultra-short-term root mean square of successive differences (RMSSD) was manually calculated to assess parasympathetic activity [33]. ECGs with non-sinus rhythm, aberrant beats, premature ventricular beats, or ectopic beats were excluded from this analysis. RMSSD was also adjusted for ECG heart rate (RMSSDc) [34].

### Holter monitoring

Each participant was referred to 24-h AECG with a Holter monitor at their affiliated heart clinic and instructed to complete a diary of activities and symptoms during the monitoring period. Measurements of rhythm, minimum HR, maximum HR, and average HR were collected for each participant. Concluding bradyarrhythmia diagnosis was obtained until the end of December 2022.

### Statistical analysis

Statistical analysis was conducted using SPSS version 28.0 statistical software (IBM Corp., Armonk, NY, USA). A Chi-square (*χ*^2^) test was used to compare nominal data. If a variable had an expected count < 5, Fisher’s exact test was used. Nominal data included variables such as prior atrial fibrillation, hypertension, and the use of prescribed medications. Due to the small size of the cohort, a Mann–Whitney *U*-test was used to compare continuous data, which included variables such as age and cerebrospinal markers. Simple linear regression was used to assess the relationship between continuous variables. A *p*-value < 0.05 was considered to indicate statistical significance.

## Results

Of the 30 enrolled participants, two later declined to perform both the MIBG scintigraphy and AECG monitoring. Of the remaining 28 subjects, 24 (85.7%) were males, and the median age was 73 (range 53–85) years. Among 25 participants who had conducted a recent MMSE test, the median score was 23 (range 15–30), with the remaining three participants having conducted either the MoCa or RUDAS. With regard to DLB, seven (25%) participants had all four core features, 12 (42.9%) had three, seven (25%) had two, and the remaining two (7.1%) participants only had one core feature along with ≥ 1 indicative biomarkers. Median disease duration (time between DLB diagnosis and study inclusion) was 6 (range 0–57) months. In total, 13 (46.4%) participants had a documented history of falling, 20 (71.4%) of recurring dizziness, five (17.9%) of palpitations, and seven (25%) of presyncope (near fainting experience) and/or syncope.

Twenty-three participants (82.1%) had a cardiac MIBG scintigraphy result indicative of cardiac sympathetic denervation with a median HMR of 1.2 (range 0.9–2.3). Median RMSSD and RMSSDc was 14.4 (range 6.9–138.7) and 17.6 (range 9.28–77.4), respectively, with four participants excluded from analysis due to heart beats not generated by sinus node depolarization.

None of the 28 participants was under current investigation due to bradycardia or had a prior diagnosis of second- or third-degree AV block or SSS. Six (21.4%) participants had prior atrial fibrillation (AF). Three participants (10.7%) were prescribed an oral beta-blocker (metoprolol), one was also prescribed digoxin and an alpha blocker (alfuzosin), and another participant was prescribed eye drops containing timolol; no participant was prescribed other class IV antiarrhythmic agents. One participant was prescribed a dihydropyridine calcium channel blocker (amlodipine), 15 (53.6%) were prescribed acetylcholinesterase inhibitors (AChEI), and eight (28.6%) were prescribed levodopa/benserazide.

**Fig. 1:**
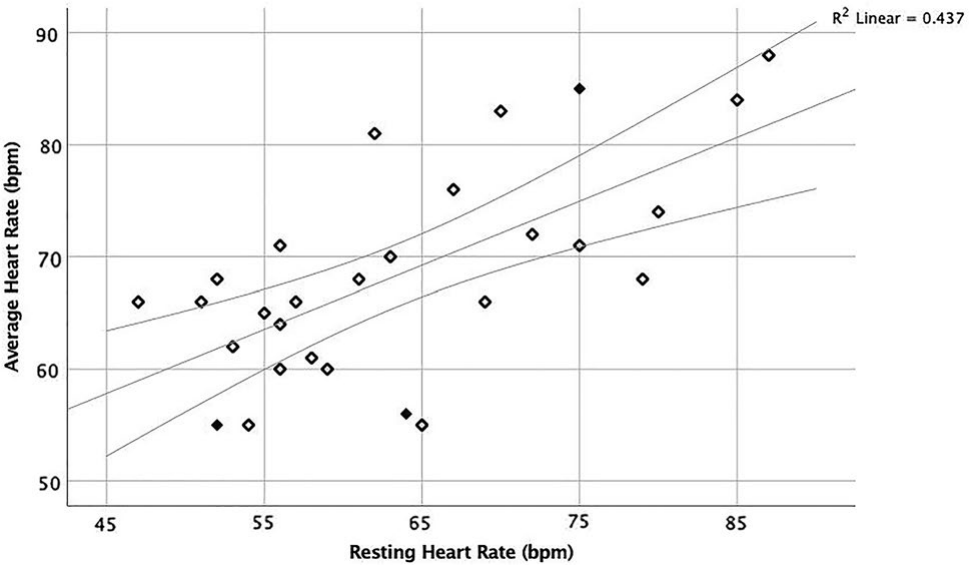
Association between average heart rate during the inclusion ambulatory electrocardiographic monitoring and heart rate after 10 min of resting. Solid symbols represent participants with subsequent sick sinus syndrome. *bpm* Beats per min

During orthostatic testing, the median resting HR was 61.5 (range 47–87) bpm, with 13 (46.4%) participants having bradycardia at rest. Seventeen (60.7%) participants had nOH. Comparisons of characteristics between participants with and without bradycardia at rest are shown in Table 1. There was an association between resting HR and average HR during AECG monitoring (standardized beta 0.661, 95% confidence interval [CI] 0.310–0.832; *p* < 0.001) (Fig. 1).

**Table 1:** Demographics and clinical characteristics of participants with and without bradycardia after 10 min of resting during orthostatic testing

Patient characteristics	Resting HR < 60 bpm (*n* = 13)	Resting HR ≥ 60 bpm (*n* = 15)	*p*-value
Age at inclusion (years)	73 (12)	76 (16)	0.786
Female gender	1 (7.7%)	3 (20%)	0.356
Education (years)	12 (8)	12 (2)	0.683
DLB disease duration (months)	6 (16.5)	2 (32)	0.274
MMSE (score) (11 vs. 14)	23 (10)	25 (8)	0.467
Core features of DLB			
RBD	9 (69.2%)	12 (80%)	0.412
Fluctuations	11 (84.6%)	10 (66.7%)	0.258
Hallucinations	8 (61.5%)	10 (66.7%)	0.544
Parkinsonism	10 (76.9%)	10 (66.7%)	0.431
Other symptoms^a^			
Palpitations	3 (23.1%)	2 (13.3%)	0.428
Falls	5 (38.5%)	8 (53.3%)	0.343
Dizziness	8 (61.5%)	12 (80%)	0.255
Presyncope/syncope	1 (7.7%)	6 (40%)	0.060
Comorbidities			
Atrial fibrillation	4 (30.8%)	2 (13.3%)	0.255
Chronic heart failure	3 (23.1%)	0	0.087
Ischemic heart disease	4 (30.8%)	2 (13.3%)	0.255
Diabetes	1 (7.7%)	0	0.464
Hypertension	6 (46.2%)	4 (26.7%)	0.249
Stroke	2 (15.4%)	1 (6.7%)	0.444
Sympathetic denervation	11 (84.6%)	12 (80%)	0.572
RMSSD (12 vs. 12)	21.2 (25.1)	9.7 (7.0)	0.007*
ECG HR (12 vs. 12)	58.5 (10.3)	72.5 (18.3)	0.003*
RMSSDc (12 vs. 12)	18.4 (19.6)	16.1 (16.9)	0.443
nOH	9 (69.2%)	8 (53.3%)	0.320
CSF biomarkers (10 vs. 13)			
Aβ42/40	0.79 (0.47)	0.94 (0.46)	0.257
Tau	257 (236)	288 (253)	0.784
Phosphorylated tau	36.5 (33)	38 (32.5)	0.738
Prescribed			
Metoprolol	2 (15.4%)	1 (6.7%)	0.444
Alfuzosin	1 (7.7%)	0	0.464
Amlodipine	1 (7.7%)	0	0.464
Digoxin	1 (7.7%)	0	0.464
Levodopa/benserazide	4 (30.8%)	4 (26.7%)	0.569
AChEI	6 (46.2%)	9 (60%)	0.362

Values in table are presented as the median with the interquartile range (IQR) in parenthesis; *n* = columnar %

*Significant difference between participants with and without bradycardia after 10 min of resting at *p* < 0.05

*HR* Heart rate, *bpm* beats per min, *MMSE* Mini-Mental State Examination, *DLB* dementia with Lewy bodies, *RBD* rapid eye movement sleep behavior disorder, *RMSSD* root mean square of successive differences corrected for heart rate*, ECG* electrocardiogram, *RMSSDc* RMSSD corrected for ECG HR, *nOH* neurogenic orthostatic hypotension, *CSF* cerebrospinal fluid, *AChEI* acetylcholinesterase inhibitor

^a^Prior to inclusion

During AECG monitoring, 26 participants had sinus rhythm, and two had atrial fibrillation. The median minimum HR was 48 (range 39–71) bpm, the median maximum HR was 110 (range 82–166) bpm, and the median average HR was 67 (55–88) bpm. Four participants (14.3%) had bradycardia for half of the monitoring period (average HR < 60 bpm), of whom all had sinus rhythm. Characteristics of participants with and without an average HR < 60 bpm are shown in Table 2. In total, five (17.9%) participants reported symptoms during the monitoring period (see Table 3).

**Table 2:** Demographics and clinical characteristics of participants with and without bradycardia during half of the monitoring period

Patient characteristics	Average HR < 60 bpm (*n* = 4)	Average HR ≥ 60 bpm (*n* = 24)	*p* value
Age at inclusion (years)	74.5 (14)	72.5 (16)	0.390
Female gender	1 (25%)	3 (12.5%)	0.481
Education (years)	14.5 (7)	12 (5)	0.262
Disease duration (years)	7 (17.75)	5 (28.5)	0.825
MMSE (score) (4 vs. 21)	26.5 (5.5)	23 (10)	0.231
Core features of DLB			
RBD	1 (25%)	20 (83.3%)	0.038*
Fluctuations	3 (75%)	18 (75%)	0.708
Hallucinations	4 (100%)	14 (58.3%)	0.149
Parkinsonism	2 (50%)	18 (75%)	0.318
Other symptoms^a^			
Palpitations	1 (25%)	4 (16.7%)	0.568
Falls	3 (75%)	10 (41.7%)	0.244
Dizziness	2 (50%)	18 (75%)	0.318
Presyncope/syncope	1 (25%)	6 (25%)	0.747
DLB disease duration (months)	7 (17.5)	5 (28.5)	0.825
Symptoms reported in Holter diary	2 (50%)	3 (12.5%)	0.135
Comorbidities			
Atrial fibrillation	2 (50%)	4 (16.7%)	0.191
Chronic heart failure	1 (25%)	2 (8.3%)	0.382
Ischemic heart disease	0	6 (25%)	0.357
Diabetes	0	1 (4.2%)	0.857
Hypertension	2 (50%)	8 (33.3%)	0.452
Stroke	0	3 (12.5%)	0.618
Sympathetic denervation	3 (75%)	20 (83.3%)	0.568
RMSSD (4 vs. 20)	31.4 (93.1)	12.6 (10.8)	0.013*
ECG HR (4 vs. 20)	50 (22.8)	67 (19.8)	0.029*
RMSSDc (4 vs. 20)	37.9 (57.9)	17.6 (13.7)	0.309
nOH	3 (75%)	14 (58.3%)	0.482
CSF biomarkers (3 vs. 20) (range)			
Aβ42/40	0.60 (0.22)	0.86 (0.82)	0.309
Tau	286 (587)	259 (545)	0.966
Phosphorylated tau	43 (48)	38 (91)	0.698
Prescribed			
Metoprolol	1 (25%)	2 (8.3%)	0.382
Alfuzosin	1 (25%)	0	0.143
Amlodipine	0	1 (4.2%)	0.857
Digoxin	1 (25%)	0	0.143
Levodopa/benserazide	1 (25%)	7 (29.2%)	0.682
AChEI	2 (50%)	13 (54.2%)	0.644

Values in table are presented as the median with the interquartile range (IQR) in parenthesis; *n* = columnar %

All participants with an average HR < 60 bpm had sinus rhythm during the ambulatory electrocardiography monitoring

*HR* Heart rate, *bpm* beats per min, *MMSE* Mini-Mental State Examination, *DLB* dementia with Lewy bodies, *RBD* rapid eye movement sleep behavior disorder, *RMSSD* root mean square of successive differences corrected for heart rate*, ECG* electrocardiogram, *RMSSDc* RMSSD corrected for ECG HR, *nOH* neurogenic orthostatic hypotension, *CSF* cerebrospinal fluid, *AChEI* acetylcholinesterase inhibitor

*Significant difference between participants with and without bradycardia during half of the monitoring period after at *p* < 0.05

^a^Prior to inclusion

**Table 3:** Self-reported symptoms during inclusion Holter monitoring period and corresponding electrocardiographic findings among all participants

Participant	Self-reported symptoms	Corresponding electrocardiographic findings
1	“Palpitations”	Premature ventricular beats
2	“Dizziness”	No electrocardiographic findings
3	“Dizziness”	No electrocardiographic findings
4^a^	“Dizziness”	Inadequate heart rate increase during physical activity
5	“Palpitations”	Sinus tachycardia

^a^Concluding sick sinus syndrome (SSS) based on inclusion Holter monitoring

By the end of December 2022, three participants (10.7%) had been diagnosed with SSS by their affiliated heart clinic. One participant was diagnosed with SSS following the inclusion AECG monitoring and received a pacemaker implant to manage associated dizziness. One was diagnosed with SSS (tachycardia–bradycardia syndrome) after follow-up AECG monitoring and was considered not to benefit from a pacemaker implant (the risk of surgery was deemed greater than the presumed gain in symptom relief) and was referred for annual follow-up AECG via the heart clinic. The associated symptom was regarded as fatigue. Additionally, one other participant was diagnosed with SSS following an episode of syncope a few months after the inclusion AECG monitoring (which at the time had not required follow-up investigation) and received a pacemaker implant to manage associated syncope. Prescribed medications had remained the same at the time of inclusion for this participant.

Following the inclusion AECG monitoring and an adjacent follow-up exercise stress test (suggestive of asymptomatic chronotropic incompetence), one other participant was assessed by the heart clinic as potentially having SSS, without a concluding diagnosis being set. This participant was to undergo follow-up AECG monitoring during 2023. Individual characteristics of the three participants with confirmed SSS and the one with potential SSS are listed in Table 4*.* None of the 28 participants was diagnosed with second- or third-degree AV block or novel AF.

**Table 4:** Clinical characteristics of participants with concluded sick sinus syndrome or potential sick sinus syndrome by the end of 2022

Participants^a^	A	B	C	D
Sick sinus syndrome	Yes	Yes	Yes	Potentially
Associated symptom	Dizziness	Fatigue	Syncope	None established
Received a pacemaker implant	Yes	No	Yes	–
Average HR (bpm)	56	55	85	60
Minimum HR (bpm)	45	40	50	44
Maximum HR (bpm)	88	120	160	82
Resting HR (bpm)^b^	64	52	75	59
No. of core features	1	3	4	2
DLB disease duration (months)	0	6	32	6
MMSE (score)	30	23	23	28
Heart/mediastinum ratio	1.2	1.1	1.1	1.1
RMSSD	21.3	21.3	N/A^d^	21.1
ECG HR	48	52	96^d^	54
RMSSDc	14.4	16.4	N/A^d^	17.3
Atrial fibrillation^c^	No	Yes	Yes	No
Hypertension	No	Yes	Yes	Yes
Chronic heart failure	No	Yes	No	No
Ischemic heart disease	No	No	Yes	No
Prescribed				
Metoprolol	No	No	Yes	No
Levodopa/benserazide	No	No	Yes	No
AChEI	No	Yes	Yes	Yes

^a^All were males and aged > 65 year at inclusion. None had diabetes or prior stroke. None were prescribed alpha blockers, digoxin, or calcium channel blockers. All had cardiac sympathetic denervation and nOH. Participant B had atrial fibrillation during the ambulatory electrocardiographic monitoring

^b^Orthostatic testing

^c^According to medical records

^d^Not available; atrial fibrillation

## Discussion

This study was conducted to explore the prevalence of undetected bradyarrhythmia in a cohort of people with clinical DLB. Our main finding was a notably high prevalence of SSS compared with estimates in the general population [15], which is in accordance with a recent clinicopathological study which found that 5.2% of patients with LBD had concurrent SSS [14]. In addition, two of the enrolled participants in our study received subsequent pacemaker implants to manage associated symptoms of SSS, suggesting that concurrent SSS might be a modifiable factor to reduce symptom burden.

It has been proposed that cardiac Lewy body pathology might manifest as atrial arrhythmias [7, 11]. The presence of α-syn has been observed in the sinus node [7], and SSS is known to be occasionally caused by infiltrative diseases, such as amyloidosis and sarcoidosis [12]. The observed high frequencies of concurrent SSS in LBD might therefore be a manifestation of cardiac Lewy body pathology of the sinus node.

An association between cardiac sympathetic denervation and cardiac α-syn pathology has been suggested [8, 9]. However, cardiac sympathetic denervation has not been examined in relation to Lewy body pathology of the cardiac conduction system. The presence of cardiac sympathetic denervation did not differ between participants with and without bradycardia at rest or with an average HR < 60 bpm. However, because 82.1% of all participants demonstrated cardiac sympathetic denervation, it may be too non-specific to reflect potential α-syn engagement of structures that decrease HR. RMSSD was significantly higher among participants with bradycardia at rest or those with an average HR < 60 bpm compared with those without. These findings could suggest that an increased or unopposed vagal activity is responsible for bradycardia [35], which might be caused by sympathetic denervation. The overall median RMSSD was similar to that of prior DLB research using 5-min ECG recordings [36]. However, after correcting RMSSD for ECG HR, there were no differences between the two groups. Furthermore, subjects with cardiac sympathetic denervation—as identified on MIBG scintigraphy—may also have undocumented parasympathetic denervation, and thus effects on resting HR may not be apparent.

Orimo et al. [37] conducted 24-h AECG and cardiac MIBG scintigraphy on 46 patients with Parkinson’s disease and found one case of non-sustained ventricular tachycardia in the patient with the lowest HMR (1.17). Although the authors speculated that severe arrhythmia might occur in cases of markedly decreased MIBG uptake, they did not observe any other severe arrhythmias among the remaining patients. In patients with chronic heart failure, there is conflicting evidence as to whether decreased HMR is associated with arrhythmia [38, 39].

The proportion of enrolled women was low compared with overall estimates of gender composition in DLB [40]. This might be due to phenotypic differences in the presentation of core DLB features among women, leading to misdiagnosis [41]. There were no statistically significant differences in demographics, cardiovascular comorbidities, prescription of negative chronotropic drugs or DLB disease duration when comparing the HR groups. Chronic heart failure, hypertension, prescription of beta-blockers, and AF were more frequent in participants with bradycardia at rest and average HR < 60 bpm compared with those without. However, ischemic heart disease and stroke were more frequent in those with an average HR ≥ 60 bpm, which might suggest that cardiovascular disease is not associated with pronounced bradycardia. We did not exclude participants with AF from enrollment due to the possibility of AF coexisting with SSS [42] (as was the case for 2 enrolled participants). Prescription of levodopa/benserazide was similar between groups, possibly because benserazide inhibits dopaminergic effects in the peripheral nervous system.

Time from DLB diagnosis to study inclusion spanned 0 to 32 months among the participants who received a SSS diagnosis, which might suggest that concurrent SSS is not affected by disease duration. However, disease duration might be unreliable in DLB research as the underlying neurodegenerative process may start several years before the onset of fully expressed core features [1, 43].

An association was found between resting HR and average HR during AECG monitoring. However, a decreased average HR might reflect physical inactivity and/or excessive sleeping during the monitoring period, which is common for people with DLB. Average HR may sometimes be analyzed during ambulatory blood pressure monitoring (which is used in certain memory clinics).

Another potential cause of bradycardia is carotid sinus hypersensitivity (CSH), which has an increased prevalence among people with DLB [44]. CSH is thought to be caused by degeneration of the medullary autonomic nuclei [45]. Additionally, CSH has been associated with SSS [46] and is usually diagnosed with monitored carotid sinus massage [44], which was not conducted in the present study.

Furthermore, since all patients with SSS had signs of noradrenergic deficiency demonstrated by cardiac MIBG scintigraphy and nOH, a combination of cardiac sympathetic denervation and baroreflex-sympathoneural dysfunction might have interfered with electrical depolarization or signal conduction of the heart.

To distinguish SSS from asymptomatic bradycardia, an association between symptoms at the time of ECG findings must be established [12]. As a result, difficulties in the self-reporting of non-specific symptoms of SSS (which may potentially mimic features of DLB) might lead to misdiagnosis. Therefore, a subsequent investigation with repeated AECG monitoring and/or exercise stress tests might become necessary [12], as demonstrated in this report. Scarce self-reporting of symptoms could be due to impaired interoception which might be caused by DLB neurodegeneration [47], and absence of symptoms is commonly observed for LBD patients with orthostatic hypotension [48, 49]. Additionally, there might be participants with continuously undetected SSS who had a normal inclusion AECG during the 24-h monitoring period. A history of falls and palpitations was more prevalent among people with an average HR < 60 bpm compared with those without, which could be due to prior arrhythmia not detected in this study.

The prescription of cholinesterase inhibitors in the present cohort was low compared with that reported in the overall Swedish DLB population (53.6% vs. 76.1%) [50]. This might be due to prior episodes of bradycardia or ECG alterations appearing as adverse drug reactions and, therefore, increased caution in the prescribing of these drugs. Such reluctance could have resulted in selection bias when enrolling participants for this study. On the other hand, the fact that the presence of cardiac devices, including pacemakers, constituted an exclusion criterion in this study can be considered a bias reducing the prevalence of arrhythmia.

It can be argued that the prevalence of bradyarrhythmia in this DLB cohort could be iatrogenic and not part of the underlying disease process, given that two of the three patients with SSS were undergoing AChEI treatment. However, in a randomized, double-blinded, placebo-controlled clinical trial (excluding patients with SSS and cardiac conduction defects) of 541 patients with Parkinson’s disease dementia, one patient (0.3%) had novel SSS diagnosed after 24 weeks of AChEI treatment compared with none receiving placebo [51]. Furthermore, in a cohort of 60 AD patients undergoing AChEI treatment, AECG revealed no cases of alarming bradycardia and no patients underwent subsequent pacemaker implantation [52], suggesting that AChEI treatment alone cannot explain our findings. Furthermore, one of the two patients who received a pacemaker after AECG monitoring in the present cohort had no history of AChEI use. Continuation of AChEI treatment was considered necessary for the patient who did not receive a pacemaker implant.

The small sample size, a disproportionate gender composition, and lack of neuropathological verification of underlying Lewy body pathology are the major limitations of our study. Future work should use larger cohorts and could include patients with other causes of dementia (e.g., AD) as control groups. Such studies could also utilize time and frequency heart rate variability domains based on longer ECG recordings. To understand further if and how Lewy body pathology might contribute to the development of arrhythmia, future clinicopathological studies should systematically examine the sinus node, cardiac conduction system, and autonomic nerves targeting the heart in relation to bradyarrhythmia diagnosis. Further research should also explore how patients with DLB and concurrent SSS (and potential caregivers) might experience daily life after a pacemaker has been implanted.

## Conclusion

This study revealed a high prevalence of previously undetected SSS in a clinical cohort of DLB patients, with two participants receiving pacemaker implants to manage associated symptoms. Further research on the prevalence and potential causes and consequences of concurrent bradyarrhythmia among people with DLB is warranted.

## Data Availability

The data generated in this study are available upon reasonable request from the correspondig author.
